# Reply to Eisenhut

**DOI:** 10.1093/infdis/jiu489

**Published:** 2014-08-26

**Authors:** Maximilian Muenchhoff, Philip Goulder

**Affiliations:** Department of Paediatrics, University of Oxford, United Kingdom

10.1093/infdis/jiu488

**Figure 1.**
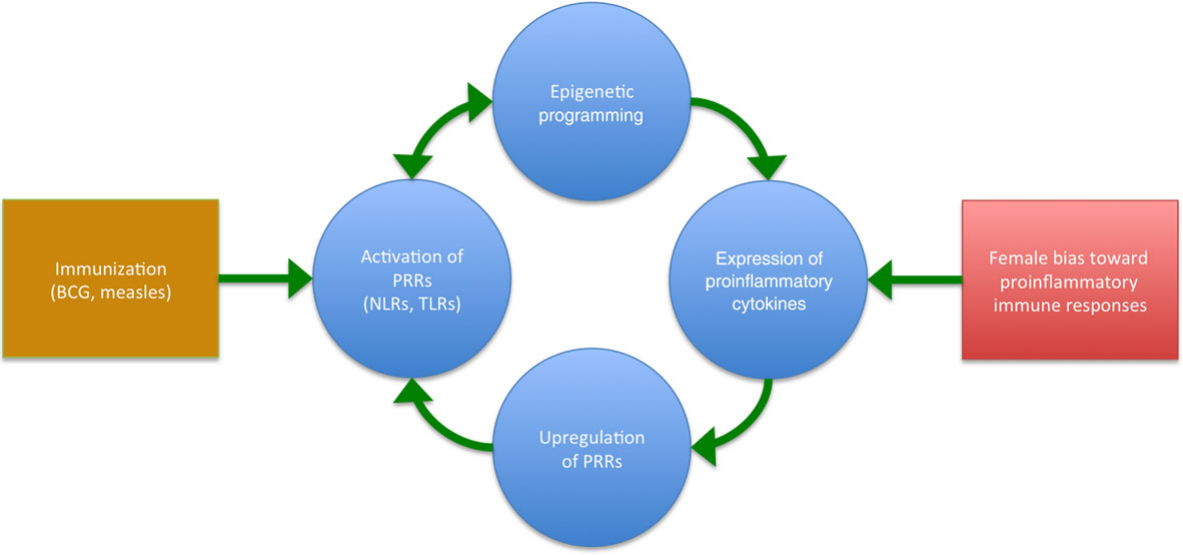
Generalized model of sexual dimorphisms in nonspecific vaccine effects. Immunization activates innate immune pathways through pattern recognition receptors (PRRs). Epigenetic programming of innate immune genes persistently enhances expression of proinflammatory cytokines. This effect of trained immunity is stronger in females because of their bias toward proinflammatory immune responses. Proinflammatory cytokines induce the expression of innate immune genes, such as those encoding PRRs, in a positive feedback loop. Abbreviations: NLRs, NOD-like receptors; TLRs, Toll-like receptors.

To the Editor—In response to our recent article on sex differences in pediatric infectious diseases [1], Eisenhut [2] highlighted the role of nucleotide binding oligomerization domain receptor 2 (NOD2) in the nonspecific (heterologous) effects of BCG immunization in reducing tuberculosis-unrelated diseases that are observed especially in females. We make 2 further points here. First, the nonspecific BCG effects are not exclusive to females, as stated by Eisenhut, but are merely more pronounced in females [3]. Second, Eisenhut's proposed mechanism for the sexual dimorphism observed in relation to these nonspecific effects of BCG immunization may be more broadly applicable to explain why heterologous vaccine effects are generally stronger in females. Heterologous effects of vaccines influence morbidity and mortality independent of the vaccine's target disease by modulating subsequent immune responses upon exposure to unrelated stimuli [3]. These nonspecific vaccine effects are largely attributed to enhanced innate immune responses through a process termed “trained immunity” [4] and are generally stronger in females [5]. Eisenhut's proposed mechanism for the stronger heterologous effects of BCG immunization observed in females [6] is based on their stronger bias towards proinflammatory T-helper type 1 responses. A positive feedback loop is suggested, in which muramyl dipeptide from BCG activates NOD2, consequently inducing nuclear factor κ-light-chain enhancer of B cells (NF-κB) activation, leading to increased interferon γ expression, which is generally higher in females, and to further upregulation of NOD2. Other proinflammatory cytokines that also signal through NF-κB, such as tumor necrosis factor α, are likely to be involved in a similar way. A proposed model of the sex-skewed nonspecific vaccine effects is shown in Figure 1.

The shift toward proinflammatory immune responses through epigenetic programming after certain immunizations, which is stronger in females, might also explain increased female mortality rates observed after subsequent vaccinations. Diphtheria–tetanus–pertussis vaccination (DPT) is associated with increased mortality rates in girls, and this effect is stronger if BCG immunization was given previously [7, 8]. A similar increase in female mortality was observed if DPT was given after a high-titer measles vaccination [9]. These observations suggest an important effect of previously administered vaccines on subsequently given immunizations, and this is likely mediated by trained innate immunity. Further studies on these important heterologous vaccine effects on trained immunity and how they differ between the sexes are therefore needed to optimize vaccination schedules and strategies.
